# A model to determine the effect of collagen fiber alignment on heart function post myocardial infarction

**DOI:** 10.1186/1742-4682-11-6

**Published:** 2014-01-22

**Authors:** Andrew P Voorhees, Hai-Chao Han

**Affiliations:** 1 Biomedical Engineering Program, UTSA-UTHSCSA 1 UTSA Circle, San Antonio, TX 78249, USA; 2 Department of Mechanical Engineering, The University of Texas at San Antonio Biomedical Engineering Program, UTSA-UTHSCSA, 1 UTSA Circle, San Antonio, TX 78249, USA

**Keywords:** Cardiac mechanics, Myocardial infarction, Collagen fiber alignment, Microstructure based mechanical model, Adverse remodeling, Anisotropy

## Abstract

**Background:**

Adverse remodeling of the left ventricle (LV) following myocardial infarction (MI) leads to heart failure. Recent studies have shown that scar anisotropy is a determinant of cardiac function post-MI, however it remains unclear how changes in extracellular matrix (ECM) organization and structure contribute to changes in LV function. The objective of this study is to develop a model to identify potential mechanisms by which collagen structure and organization affect LV function post-MI.

**Methods:**

A four-region, multi-scale, cylindrical model of the post-MI LV was developed. The mechanical properties of the infarct region are governed by a constitutive equation based on the uncrimping of collagen fibers. The parameters of this constitutive equation include collagen orientation, angular dispersion, fiber stiffness, crimp angle, and density. Parametric variation of these parameters was used to elucidate the relationship between collagen properties and LV function.

**Results:**

The mathematical model of the LV revealed several factors that influenced cardiac function post-MI. LV function was maximized when collagen fibers were aligned longitudinally. Increased collagen density was also found to improve stroke volume for longitudinal alignments while increased fiber stiffness decreased stroke volume for circumferential alignments.

**Conclusions:**

The results suggest that cardiac function post-MI is best preserved through increased circumferential compliance. Further, this study identifies several collagen fiber-level mechanisms that could potentially regulate both infarct level and organ level mechanics. Improved understanding of the multi-scale relationships between the ECM and LV function will be beneficial in the design of new diagnostic and therapeutic technologies.

## Background

Coronary heart disease is the leading cause of death, accounting for over 400,000 lives in the United States every year [1]. Blockage of the diseased coronary arteries leads to myocardial infarction (MI). Post-MI, the left ventricle (LV) undergoes a complex remodeling process that results in the formation of a scar or infarct. The increased stiffness and diminished contractility of the scar reduce LV function and can lead to heart failure [2]. The decrease in LV pump function has been correlated to the size of the infarct, but infarct size alone cannot explain patient outcome [3]. One key factor affecting LV function is the mechanical properties of the scar tissue. Increased infarct stiffness reduces inflation under systolic pressures, but also impairs filling of the LV during diastole [4]. Therefore, it is imperative to elucidate the relationship between scar composition and mechanics in order to identify the properties that best preserve LV function.

Cardiac scar tissue is composed of collagen fibers, the primary determinant of the mechanical properties of the scar, as well as cells and other extracellular matrix proteins which are considered to be a ground substance [2]. Thus, cardiac scar tissue demonstrates anisotropic, non-linear material behavior [5]. Naturally, the density and alignment of collagen fibers affect the mechanical properties of scar tissue and thus the wall stress, with scar tissue demonstrating a high stiffness along the collagen fiber direction and a lower stiffness in the cross-fiber directions [6]. Furthermore, it has been shown that collagen deposition during remodeling is highly controlled by the local stresses or strains in the tissue [7-9]. In arteries it has been shown that the angle of mean collagen fiber alignment adapts to optimize a tissue’s load-bearing ability which leads to fibers that are aligned in a direction in between the directions of the two principal stresses [10]. In fact, recent studies by Fomovsky et al. have shown that mechanical cues are the primary determinant of collagen alignment [11]. Therapeutic strategies that aim to limit infarct expansion by reducing local stress are currently under development [12,13], including efforts to anisotropically reinforce the infarct region [14]. Thus, it is of clinical interest to determine the relationship between collagen alignment, scar tissue mechanics, and LV function, in order to find a possible alignment that would better preserve LV function.

The objective of this study was to illustrate how collagen alignment affects LV function post-MI using a simple, thin-walled, cylindrical LV model with a scar region governed by a collagen fiber based constitutive equation. The cylindrical model is based on a concept, previously presented by Han et al. [15] and Oshinski et al. [16], to predict ejection fraction from MRI images, and the collagen fiber-based constitutive equation was based on the work of Grytz and Meschke [17]. This simple multi-scale model allows us to focus on identifying the properties of the collagen matrix that primarily determine LV function post-MI.

## Main text

### Methods

#### Constitutive model

**Figure 1:**
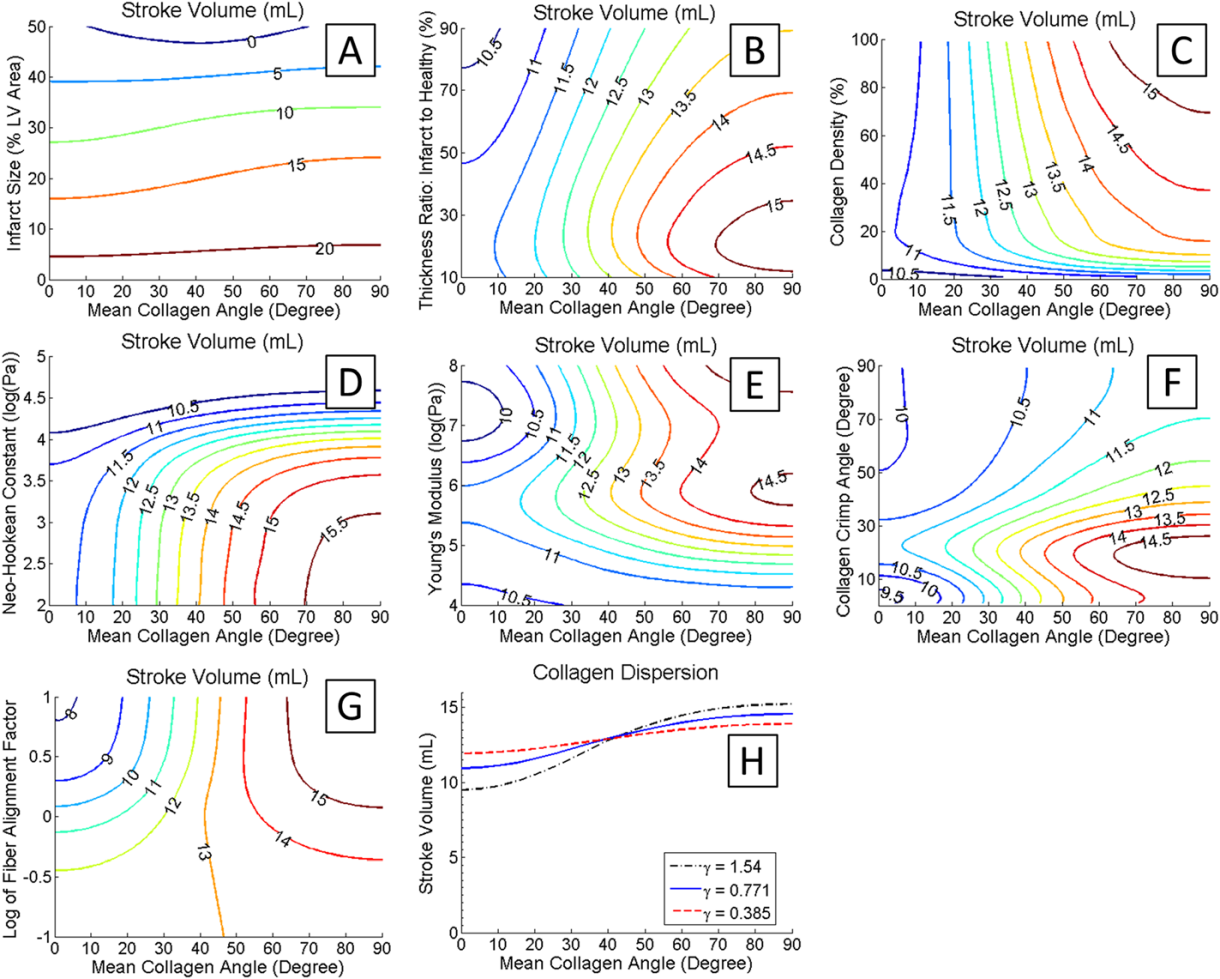
**Schematic of the four-region cylindrical model.** The heart wall was modeled as blocks of membrane of normal contraction (R: remote) and scar (S) with roman numeral supercripts used to denote the three different remote regions. Labels are given to denote the circumferential length (*L*), longitudinal height (*H*), and wall thickness (*T*) of the regions.

The LV was modeled as a cylindrical membrane formed by folding a planar sheet consisting of three normal, healthy myocardial regions and a collagenous scar region into a cylinder (Figure 1). The mid-wall circumference of the cylinder was taken to be the length of the planar sheet in the circumferential direction. The healthy LV regions were modeled as sheets with nine layers having myocyte fiber angles ranging from −50 to +50 degrees relative to the circumferential direction. While reports of myocyte rotation in the literature are somewhat variable between species, location in the ventricle, and measurement technique [18-20], the distribution parameters chosen provide a reasonable physiological approximation. The healthy region was governed by a Fung-type exponential strain energy density equation [21,22]:

(1) $$W=\frac{1}{2}c\left(e^{Q}-1\right),$$

with

(2) $$Q=b_{\mathrm{ff}}{E_{\mathrm{ff}}}^{2}+b_{\mathrm{xx}}\left({E_{\mathrm{cc}}}^{2}+{E_{\mathrm{rr}}}^{2}\right)+b_{\mathrm{fx}}\left({E_{\mathrm{fc}}}^{2}+{E_{\mathrm{cf}}}^{2}\right).$$

Where *c* is a material constant*, b*_*ff*_*, b*_*xx*_*,* and *b*_*fx*_ are non-dimensional material constants and *E*_*ff*_, *E*_*cc*_, *E*_*rr*,_*E*_*fc*_, and *E*_*cf*_ are the components of the Green strain tensor. The subscript *f* denotes the fiber direction, the subscript *c* denotes the in-plane cross fiber direction, the subscript *r* denotes the radial (out-of-plane) direction and the subscript *x* denotes either the cross-fiber or radial direction. The Fung-type constitutive equation has been widely used in soft tissue biomechanics and is still commonly used for modeling myocardial tissue [23,24]. Since the collagen density of the healthy tissue is much lower in the healthy regions of the myocardium compared to the scar region, no explicit model of collagen is included for these regions. Cauchy stresses were determined from the strain energy density functions following [25],

(3) $$\left[\sigma \right]=-p\left[I\right]+2\left[F\right]\cdot \frac{\partial W}{\partial \left[C\right]}\cdot {\left[F\right]}^{T},$$

where *p* is a Lagrange multiplier used to enforce incompressibility, [***I***] is the identity matrix, [***F***] is the deformation gradient, and [**C**] is the right Cauchy-Green deformation tensor.

Contraction of the healthy tissue was modeled using the elastance model of active cardiac contraction proposed by Guccione et al. in the form of [26,27],

(4) $$\sigma_{\mathrm{active}}={\sigma^{0}}_{\left(l_{s}\right)}\;\cdot \;\left(1-\mathrm{co}{\mathrm{s\omega}}_{\left(\;l_{s},t\right)}\right)$$

where *l*_*s*_ and *t* are the the sarcomere length and time during a cardiac cycle, respectively. ω is a function of the sarcomere length and time. σ^0^ is the peak contraction stress that depends on cellular calcium concentration and sarcomere length [27]. The total stress in the healthy regions is calculated as the sum of the active stress which is non-zero solely along the direction of myocyte orientation, and the passive Cauchy stress tensor. The individual layers of the healthy tissue were assumed to have the same planar deformation (i.e. the transmural shear deformation was ignored).

**Figure 2:**
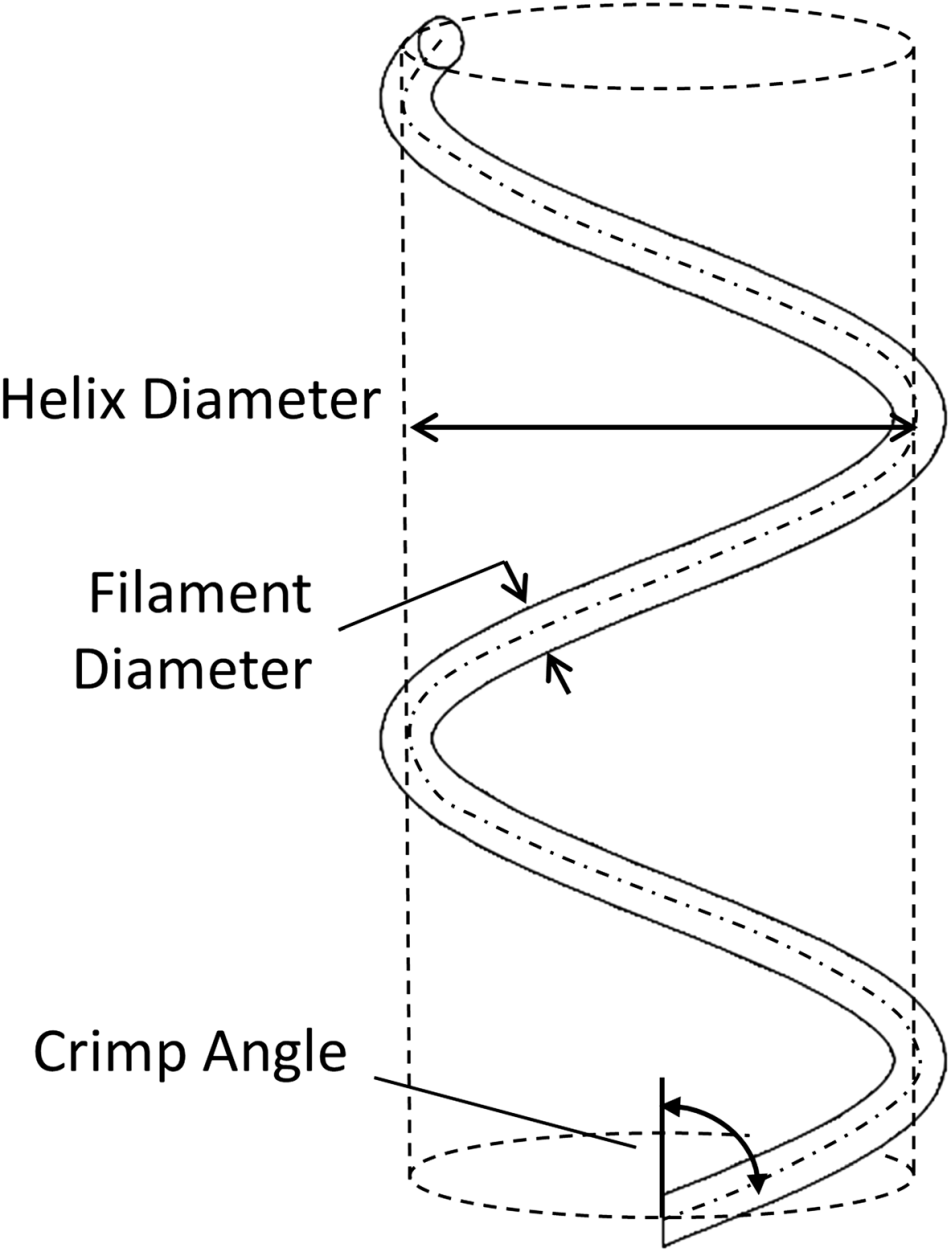
**Schematic of crimped collagen fiber.** The collagen filament coils around a central axis that is aligned in the fiber direction. The diameter of the filament, the diameter of the fiber helix, and the crimp angle are illustrated.

The scar tissue was modeled as a thin sheet composed of a distribution of collagen fibers embedded within a ground matrix. The mechanical properties of the scar region were governed by the microstructure-based nonlinear model proposed by Grytz and Meschke [17,28]. Briefly, the stretch of collagen fibers was modeled as the uncrimping of a coiled collagen filament. This is similar to the manner in which a spring uncoils as it is stretched. The strain energy density function for the collagen fiber component of the tissue, *W*_*col*_, was calculated by integrating the first Piola-Kirchoff stress, *P*_*col*_ which is a function of the stiffness of the collagen filament, *E*_*col*_, the ratio of the fiber diameter, *D*_*col*_, to the filament diameter, *d*_*col*_, and the initial crimp angle, *θ*_*o*_*,* (a measure of how tightly coiled the fiber is, with 0° representing a fully straightened filament and 90° representing a filament that has been compacted into a disc, see Figure 2) over the deformation of the fiber, *λ*.

(5) $$W_{\mathrm{col}}\left(\lambda \right)=\int_{1}^{\lambda }P_{\mathrm{col}}\left(\lambda ,E_{\mathrm{col},}\frac{D_{\mathrm{col}}}{d_{\mathrm{col}}},\theta_{o}\right)d\lambda$$

where λ depends both on the probability density function of the collagen fiber alignment, *ρ*, and the right Cauchy-Green deformation tensor [**C**] [17,28]. The ground matrix was modeled as an isotropic, neo-Hookean material with a strain energy density

(6) $$W_{g}=c_{g}\left(I_{1}-3\right)$$

where *c*_*g*_ is a material constant and *I*_1_ is the first invariant of the right Cauchy-Green deformation tensor. The total strain energy density, *W*, was calculated from the volume weighted summation of the strain energy densities of the collagen fibers, *W*_*col*_, and the ground substance *W*_*g*_,

(7) $$W=\left(1-\omega_{\mathrm{col}}\right)W_{g}+\omega_{\mathrm{col}}W_{\mathrm{col}},$$

where *ω*_*col*_ is the collagen volume fraction. For a detailed description of the calculation of *W*_*col*_ please refer to the original work by Grytz and Meshke [17,28]. Accordingly, the Cauchy stresses were determined from this strain energy density functions using Eq. (3).

### Boundary conditions and equilibrium equations

For clarity, regions will be referred to as R^I^, R^II^, R^III^, representing the healthy, remote regions, and S representing the scar region (Figure 1). In order to solve for the deformed volume of the LV it is necessary to establish boundary conditions and equilibrium equations to relate deformation to the applied loads. The base of the LV is fixed in the longitudinal direction, but not in the radial or circumferential directions. Further, it is assumed that the LV will remain cylindrical throughout deformation. Given this boundary condition and the assumptions of thin walls and incompressibility, the deformation gradient, [***F***], for each region has only 3 unique components:

(8) $$\left[F\right]=\left[\begin{array}{ccc} \lambda_{\theta } & \kappa_{\mathrm{\theta z}} & 0\\ 0 & \lambda_{z} & 0\\ 0 & 0 & \frac{1}{\lambda_{\theta }\lambda_{z}} \end{array}\right].$$

The components *λ*_*θ*_ and *λ*_*z*_ represent the stretch ratios in the circumferential and longitudinal directions respectively and *κ*_*θz*_ represents the shear deformation. Since uniform stress distributions over each region are assumed, internal forces can be directly related to the Cauchy stress through the definition [25]

(9) $$q_{i}=n_{i}a_{i}\cdot \left[\sigma \right]\;\mathrm{for}\;i=\theta ,z,$$

where ***n***_***i***_ is the normal to the *i*^th^ surface and *a*_*i*_ is the surface area of the *i*^th^ surface. The internal forces must balance the forces generated by the internal pressure. Under the assumptions of the Law of Laplace for thin-walled closed-end, cylindrical pressure vessels the magnitude of the internal forces are

(10.a) $${||q||}_{\theta }=\mathrm{Prh},$$

(10.b) $${||q||}_{z}=\mathrm{\pi P}r^{2},$$

where ***q***_***θ***_ and ***q***_***z***_ are the internal circumferential and longitudinal forces, *P* is the pressure, *r* is the deformed radius of the LV, and *h* is the deformed longitudinal height of the LV.

**Figure 3:**
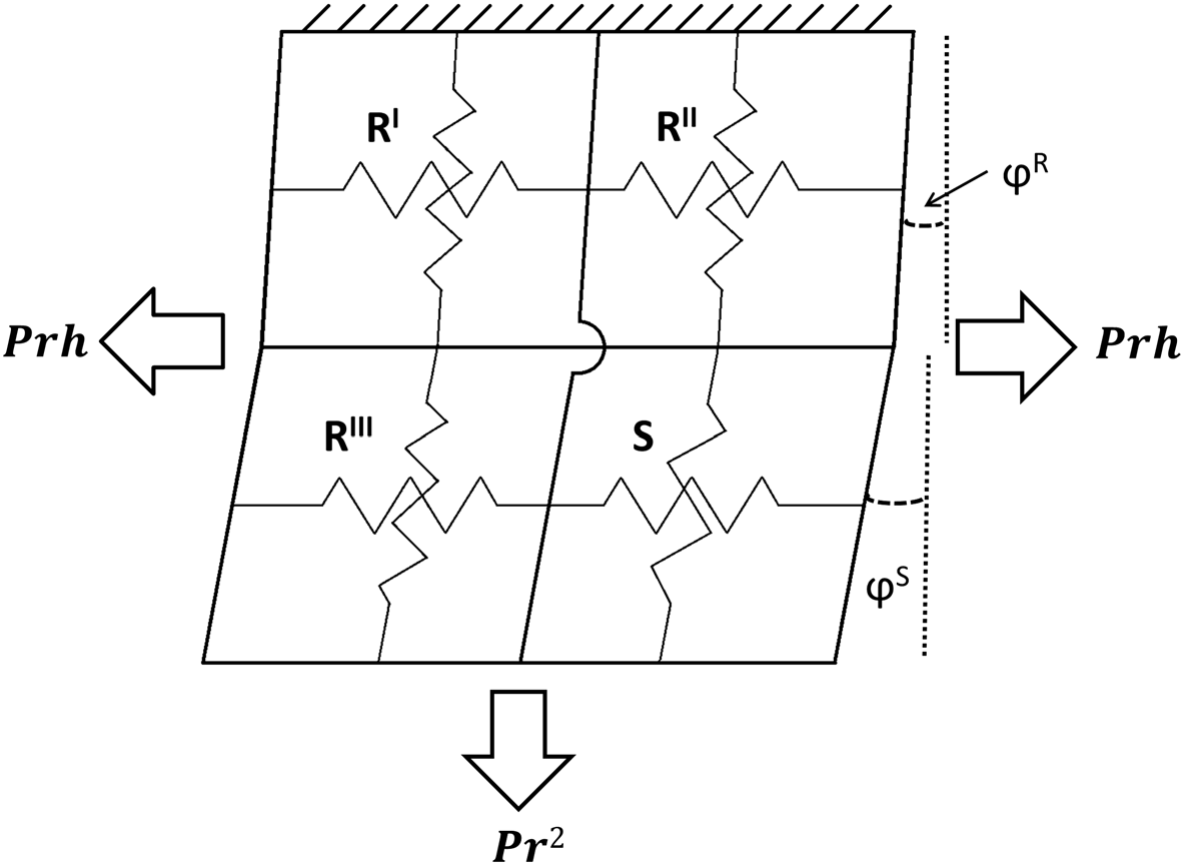
**Schematic of force division and balance.** The LV is modeled as a system of springs with a longitudinal and circumferential spring for each of the four regions. Springs shown in parallel share displacements and springs shown in series share forces. The applied forces are calculated from the Law of Laplace.

To model the coupling of the regions and the division of load amongst adjoining regions, the LV wall was modeled as a 2-D system of springs (Figure 3). Based on this arrangement, the internal loads described in Eq. (10) are shared as

(11.a) $${||q||}_{\theta }=||{q_{\theta }}^{I}||+||{q_{\theta }}^{\mathrm{III}}||=||{q_{\theta }}^{\mathrm{II}}||+||{q_{\theta }}^{S}||=\mathrm{Prh},$$

(11.b) $$||q_{z}||=||{q_{z}}^{I}||+||{q_{z}}^{\mathrm{II}}||=||{q_{z}}^{\mathrm{III}}||+||{q_{z}}^{S}||=\mathrm{\pi P}r^{2}.$$

The spring system model was also used to describe which regions share displacements. The deformations are described by the components of the deformation gradient tensor, [*F*], as seen in Eq. (8). In the circumferential direction, regions R^I^ and R^III^ are fixed in parallel so that they will always have the same circumferential deformation *λ*_*θ*_^*R*^. The same goes for regions R^II^ and S which share circumferential deformation *λ*_*θ*_^*S*^. In the longitudinal direction, regions R^I^ and R^II^ are fixed in parallel so that they will share longitudinal deformation *λ*_*z*_^*R*^ and shear deformation *κ*_*θz*_^*R*^. The same is true for regions R^III^ and S that share *λ*_*z*_^*S*^, and *κ*_*θz*_^*S*^. From Eq. (11) a full set of equilibrium equations can be derived which in conjunction with the constitutive equations can be solved numerically to determine the unknown displacements. A more complete derivation appears in the appendix.

### Numerical solution methods

The LV volume contained by each region was determined independently as:

(12) $$V^{i}=\left(\frac{l^{i}\cdot r}{2}-\frac{l^{i}\cdot t^{i}}{2}+\frac{l^{i}\cdot {\left(t^{i}\right)}^{2}}{2r}\right)\cdot h^{i},$$

where, *r* is the radius of the LV, and *l*^*i*^, *h*^*i*^, and *t*^*i*^ are the deformed length, height and thickness of each of the four regions R^I^, R^II^, R^III^ and S assuming that the deformed LV shape remains cylindrical. The total volume of the ventricle was determined by combining the component volumes. All numerical operations were carried out using Matlab (Mathworks, Natick, MA). An iterative solver based on Newton’s method with derivatives calculated by complex Taylor series expansion was used to simulate diastolic filling from 0 to an end diastolic pressure of 1.3 kPa (9.8 mm Hg) over 100 pressure increments. Convergence testing showed that end-diastolic volumes calculated using 50 pressure increments were only 0.1% less than those calculated using 100 increments. To simulate systole, 10 points on the end-systolic pressure volume relationship (ESPVR) curve were calculated using a Newton’s method solution technique to solve the force balance equations of Eq. (11) with the added time-varying myocyte contraction forces and an added isovolumic constraint so that pressure could be calculated as a function of time. Each systolic contraction was calculated using 100 time points. From this ESPVR the final systolic volume was taken to be the point on the curve at 13 kPa (98 mm Hg). Stroke volume (SV) and ejection fraction (EF) were then calculated from the end diastolic and systolic volumes.

### Numerical examples

**Figure 4:**
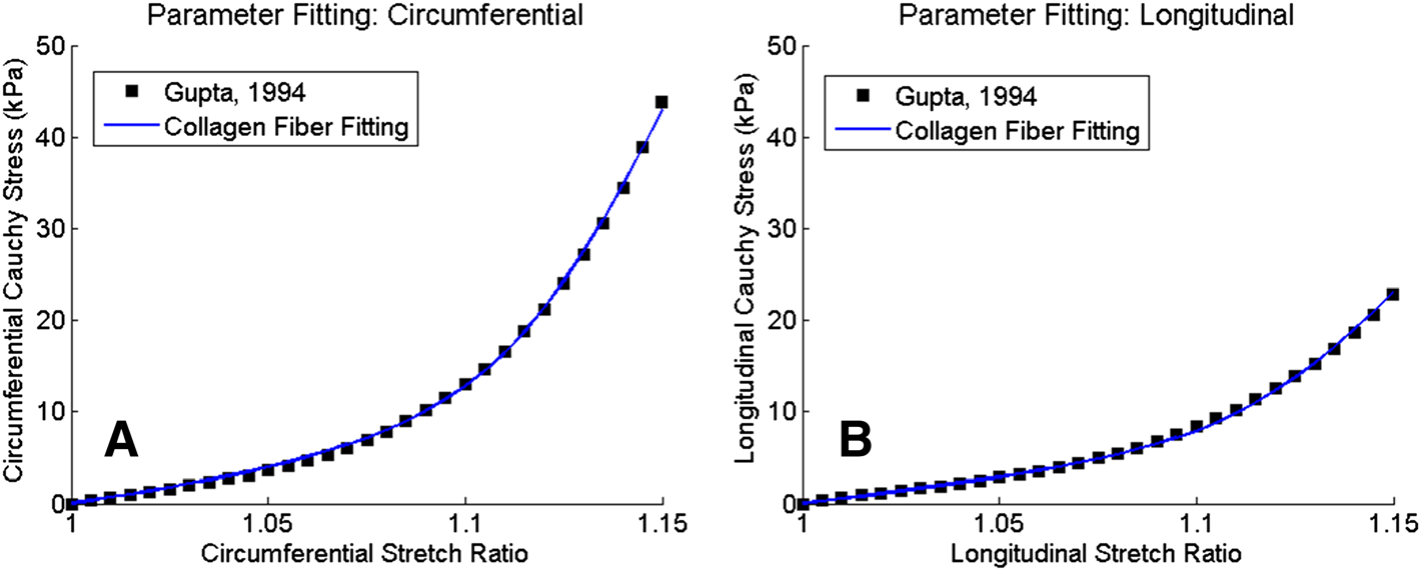
**Fitting of material properties to experimental data.** The properties of the scar tissue were fit to experimental data obtained by Gupta, et al. 1994 for a 6-week old porcine infarct. **(A)** The fitting to the circumferential stress resulted in an R^2^ value of 0.996. **(B)** The fitting to the longitudinal stress resulted in an R^2^ value of 0.994.

A set of typical material properties and geometric dimensions was chosen as an example problem for our simulation. LV dimensions were chosen so as to roughly mimic the dimensions of a canine left ventricle [29]. Total LV height was chosen to be half of the mid-line circumference. The thickness of the infarct was chosen to be half the thickness of the healthy region as well as half of the total circumference and half of the total height. In this configuration the scar represents 25% of the total myocardial area representing a moderately sized scar. The final dimensions were chosen so that the LV volume in the undeformed state matched that reported by Fomovsky et al. [24]. The material constants of the Fung equation were taken from Fomovsky et al.[24], with incompressibility assumed. The properties governing myocyte contraction were the same as those reported by Guccione, and an initial sarcomere length of 1.65 μm was chosen as this resulted in a baseline ESPVR that roughly matched the baseline case reported by Fomovsky et al. [24]. The collagen fibers of the scar region were aligned following a Von Mises distribution wrapped between −90° and +90°:

(13) $$\rho \left(\varphi \right)=\frac{\exp\left(\gamma *\cos\left(2\varphi \right)\right)}{\pi \;I_{o}\left(\gamma \right)}$$

where *γ* is a fiber alignment factor such that high values of *γ* correspond to highly aligned fibers, and *I*_*o*_ is the modified Bessel function of order zero. The properties of the scar region were determined by assuming a collagen fiber density of 40% and a circumferential mean fiber alignment and fitting the remaining parameters (crimp angle, young’s modulus, neo-Hookean constant, ratio of helix diameter to filament diameter, and fiber alignment parameter) to the stress strain relationship reported by Gupta et al. [5] for a 6 week old infarct in a porcine model. A circumferential alignment was chosen as this was the axis of the largest stress under equibiaxial stretch in the experimental data. The values for these parameters are listed in Table 1. Least-square fitting was performed using a gradient based method and the R^2^ value was 0.996 for the circumferential stress and 0.992 for the longitudinal stress (Figure 4). Using the properties described in Table 1 as a reference point, parametric studies were run to determine the effects of varying the mean collagen alignment angle, collagen density, collagen fiber alignment, collagen fiber stiffness, collagen crimp angle, scar size, and scar thickness. Additionally, a baseline case with 4 healthy regions was run for comparison.

**Table 1:** Parameters of the example infarcted LV model

**Geometry**	
Total undeformed LV length	10 cm
Total undeformed LV height	5 cm
Undeformed thickness of healthy regions	0.8 cm
Undeformed thickness of scar region	0.4 cm
**Mechanical properties of healthy regions**	
Fung constant (c)	880 Pa
Fung constant (b_cc_)	18.5
Fung constant (b_xx_)	3.58
Fung constant (b_xc_)	1.63
Number of layers	9
Myocyte fiber angle range	−50 to 50
**Mechanical properties of scar region**	
Collagen crimp angle (θ_o_)	25.5°
Ratio of collagen helix diameter (D_col_) to filament diameter (d_col_)	2.39
Young’s modulus of collagen (E_col_)	1.16 MPa
Neo-Hookean constant for ground material (c_g_)	5.72 kPa
Fiber alignment factor ( γ )	0.771
Volumetric collagen fiber fraction (ω_col_)	0.4

## Results

**Figure 5:**
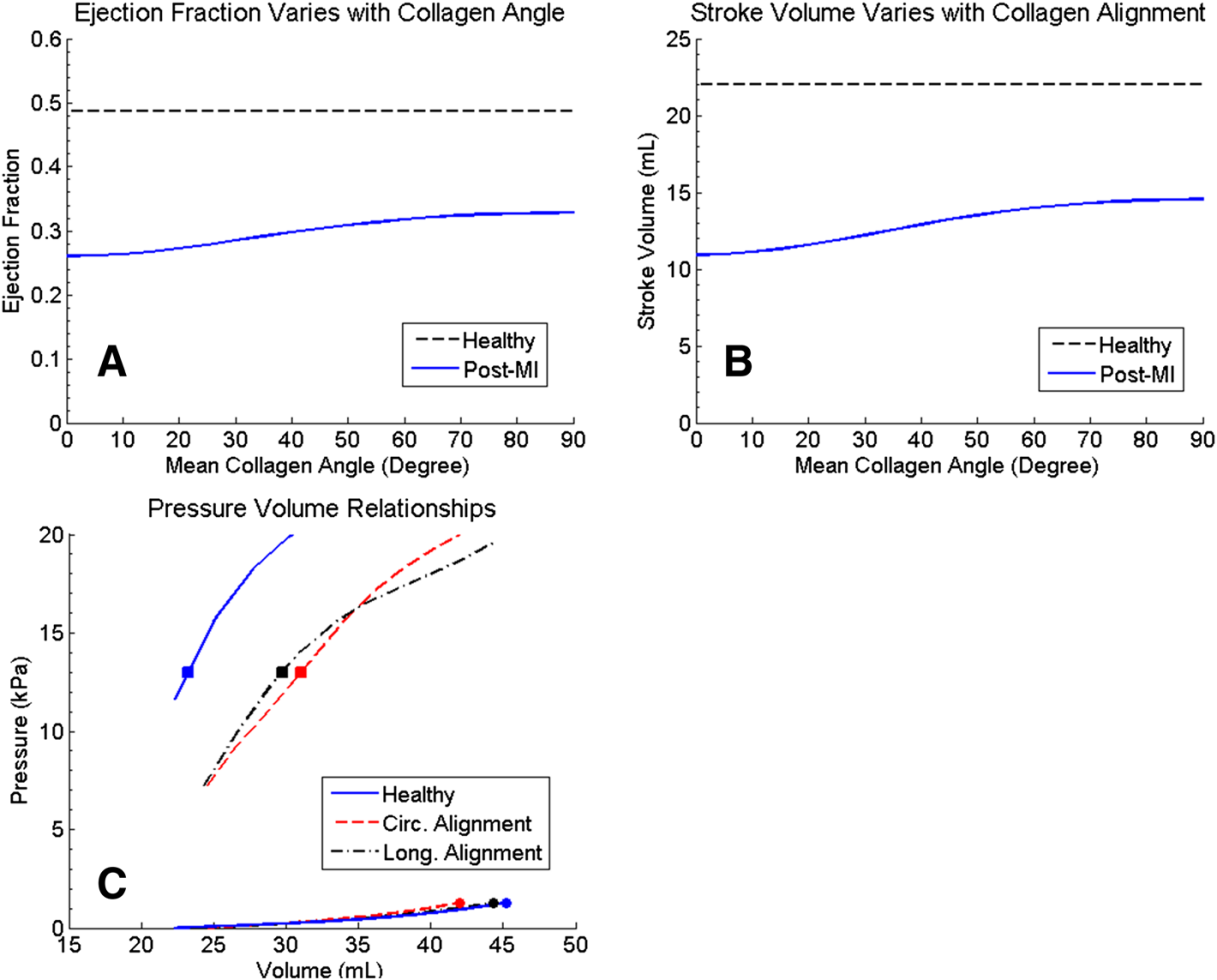
**Cardiac function of an example infarcted LV.** Changes in EF **(A)** and SV **(B)** with the mean alignment angle of the collagen in scar tissue. Dotted lines are the baseline values corresponding to the healthy LV. Both EF and SV were maximized when collagen fibers were aligned longitudinally. The pressure volume relationships **(C)** show that SV is reduced through both reduced diastolic filling and a right shift in the ESPVR. Longitudinal alignment of fibers reduces both of these effects.

The SV and EF of the baseline (pre-MI) case were 22.0 mL and 0.486 respectively. In order to investigate the effect of varying the mean fiber angle, an example infarcted LV was run 19 times with the mean fiber angle ranging from 0° (circumferential alignment) to 90° (longitudinal alignment). Simulation results showed that SV and EF increased with fiber angle and the maximum SV and EF were achieved when the fibers were aligned at 90° (Figure 5A-B). SV and EF were reduced compared to baseline through lowered diastolic filling and a right shift of the ESPVR (Figure 5C). Longitudinally aligned fibers improved diastolic filling when compared to circumferentially aligned fibers. This extra diastolic deformation also allowed myocytes in the healthy regions to generate more contractile force which slightly reduced end-systolic volume as seen in the ESPVR’s in Figure 5C. At angles intermediate to 0° and 90°, SV and EF rise steeply from around 20° to 60° before plateauing.

**Figure 6:**
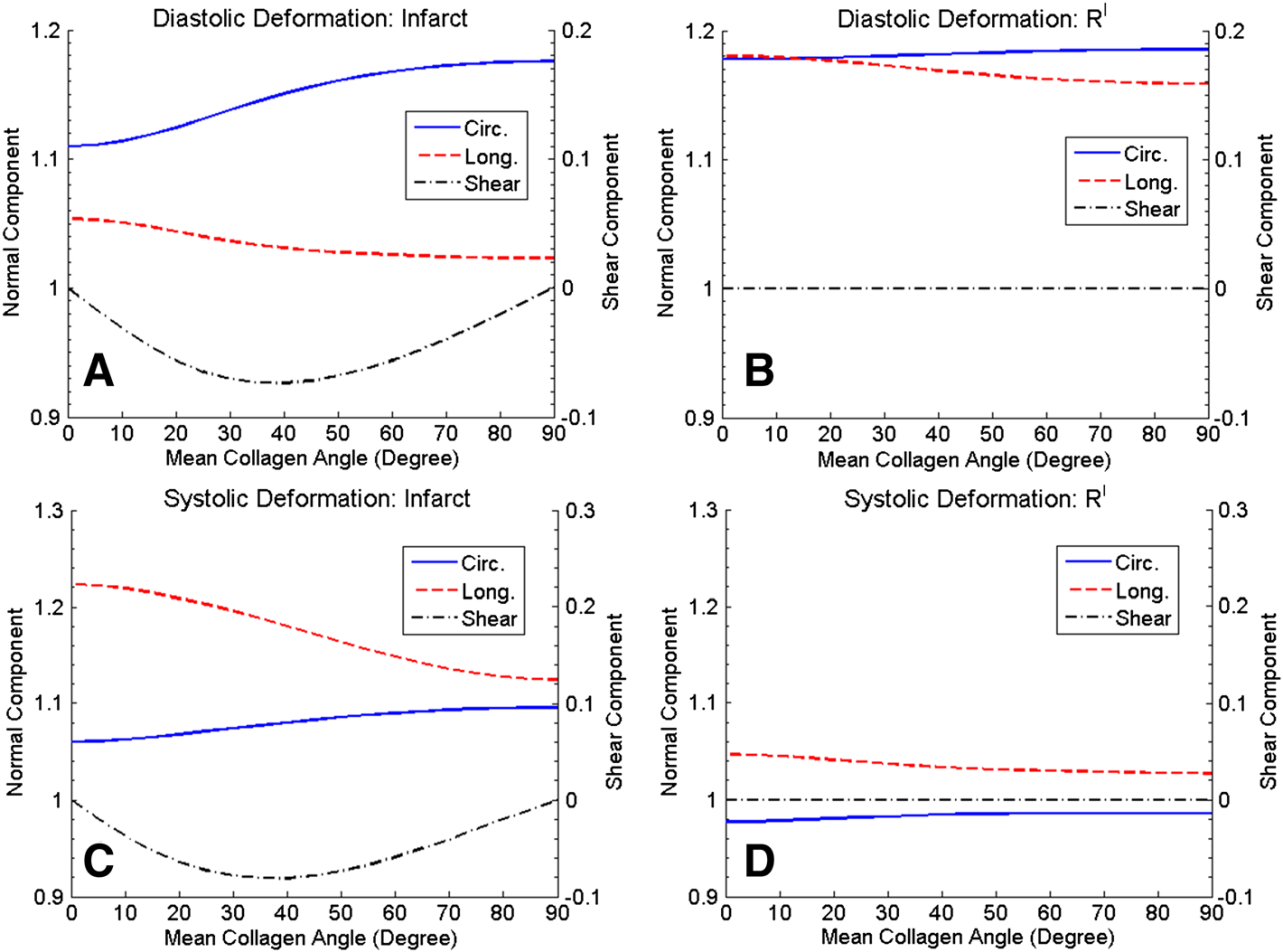
**Deformation and stress.** Diastolic deformation of the scar **(A)** and remote region R^I^**(B)** and systolic deformations of the scar **(C)** and R^I^ regions **(D)** plotted as functions of mean collagen alignment angle. The normal components of the deformation gradient (circumferential and longitudinal) are shown on the left vertical axis and the shear component is shown on the right axis.

The deformations of the scar and remote regions (S and R^I^), as measured by the components of the deformation gradient (see Eq. (8)), were also examined as the mean collagen angle was varied (Figure 6). When collagen aligns towards the longitudinal direction (90°), the circumferential deformation of the scar region increased while the longitudinal deformation at end-diastole slightly decreased (Figure 6A). Longitudinal fiber alignment also reduced the longitudinal deformation of the R^I^ region while having only a slight effect on the circumferential deformation at end-diastole (Figure 6B). At end-systole, longitudinal fiber alignment greatly reduced longitudinal deformation in the scar region while only slightly increasing the circumferential deformation (Figure 6C). Longitudinal fiber alignment reduced longitudinal deformation in the remote R^I^ region but also slightly increased the circumferential deformation at end-systole (Figure 6D).

**Figure 7:**
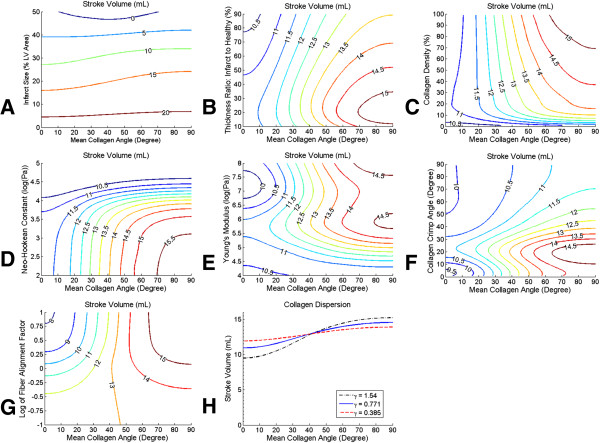
**Parametric study of SV.** Contour plots of stroke volume are presented with collagen alignment angle on the x-axis and one of the following parameters of interest on the y-axis. **(A)** Infarct size, presented as percent area of total LV area. **(B)** Wall thickness, presented as the ratio of the thickness of the infarct region to the thickness of the remote region in percent. **(C)** Collagen density presented as the percent of scar volume. **(D)** The log of the neo-hookean constant for the ground material of the infarct in log(Pa). **(E)** The log of the Young’s modulus of the collagen filament in log(Pa). **(F)** The initial crimp angle of the collagen filament in degree. **(G)** The log of the fiber alignment factor γ. **(H)** To aid in the interpretation of the contour plots, a plot of stroke volume vs. mean collagen alignment angle is presented for three levels of the fiber alignment factor γ.

Several parametric variations in material properties and geometry were simulated and are presented as contour plots (Figure 7). Increasing the size of the scar (as measured by the percentage of the total LV area greatly reduced SV (Figure 7A). The effect of increased longitudinal alignment was greatest for scars that were about 20-30% of the total LV area. Reducing the thickness of the scar tended to improve SV by allowing for greater diastolic deformation (Figure 7B). However, decreasing scar thickness below 20% of the thickness of the healthy regions resulted in decreased SV. Increasing the collagen fiber density improved SV for longitudinally aligned fibers, due primarily to increased diastolic deformation in the cross-fiber direction (Figure 7C). For circumferential alignments, increased collagen density only slightly reduced stroke volume. Increasing the neo-Hookean constant governing the ground substance of the scar region reduced the SV (Figure 7D). This effect was larger for longitudinally aligned fibers. When the Young’s modulus of the filaments was low, SV was correspondingly low (Figure 7E). Optimal stroke volumes were found when fibers were aligned longitudinally and the Young’s modulus was about 1 MPa. Increasing the Young’s modulus to 10 MPa tended to reduce SV, however increasing the modulus further to about 100 MPa increased the SV. Varying the initial crimp angle revealed that optimal SV occurred at around 20 degrees (Figure 7F). Reducing the alignment factor *γ*, a change that represents a more disperse distribution of fibers, increased the SV when the mean angle was near 0° but reduced the SV when the angle was near 90° (Figure 7G and H).

## Discussion

We developed a cylindrical LV model that links the wall stress and pumping function of the dysfunctional LV to the density and alignment of collagen fibers in the scar tissue. Using this model, we examined the effect of collagen alignment and density on LV function post-MI. Our results demonstrate that the orientation of collagen fibers in the scar region of the myocardium has a large influence on the performance of the LV. It was found that longitudinal fiber alignment limits scar deformation during systole and maximizes LV performance.

The current model clearly demonstrates how the mechanics of the healthy contractile tissue are coupled to the properties of the collagen network in the scar region. The conclusion of longitudinal aligned fibers maximizing the LV function agrees with reports in the literature [24]. This study also ellucidates the mechanism behind this improvement. The results suggest that rather than increased longitudinal stiffness improving function it is actually increased circumferential compliance that has the largest benefit. Increased circumferential compliance allows for the greatest diastolic filling, which in turn allows for greater contractile ability through the Frank-Starling mechanism. It is worth noting that an infarct that is too compliant may eventually lead to reduced systolic function as it inflates under systolic pressure. Examining the ESPVR’s in Figure 5C, it is seen that the longitudinal and circumferential alignment curves cross once the pressure exceeds about 16 kPa indicating that the increased circumferential compliance is leading to increased infarct bulging under high pressures.

While the parametric studies demonstrated that smaller scars preserve LV function they also revealed that for certain scar sizes, thinner scars increase LV function, a far less intuitive conclusion. This is due to the fact that thinner scars allow for more diastolic filling. There is a limit to this though, as scars with an infarct thickness to remote thickness ratio of less than 20% had a reduced SV due to increased systolic bulging. We do not endorse infarct thinning as an advantageous phenomenon as it increases the risk of cardiac rupture and infarct expansion.

The finding that increased collagen density has a positive effect on SV for longitudinally aligned fibers but very little effect for circumferential alignments is explained by the fact that as more collagen fibers replace the ground substance, the tissue becomes more compliant in the cross-fiber direction but stiffer in the direction of the fibers, which leads to increased diastolic filling. The importance of increased compliance is further seen in the findings that increasing the neo-Hookean constant decreased SV. One important takeaway from the parametric studies is that there appears to be an ideal stiffness at which diastolic filling is increased but systolic contraction is not greatly impaired. This is seen in the results for the infract thickness, Young’s modulus, and initial crimp angle. It is also interesting that the fitted values we found for the Young’s modulus and the crimp angle of porcine scar tissue appear to be near these ideal stiffness regions. The finding that increased fiber dispersion is beneficial for circumferentially aligned fibers but detrimental for longitudinally aligned fibers further highlights the importance of scar anisotropy in preserving ventricular function.

This work was strongly motivated by the work from the Holmes group [24]. They studied the effect of scar anisotropy on LV function using a finite element model with mechanical properties of both the healthy and infarct regions described by a Fung-type constitutive equation. By comparing isotropic scars, longitudinally stiff scars, and circumferentially stiff scars, they found that SV would be maximized when the scar tissue was stiffer in the longitudinal direction than in the circumferential direction. Fomovsky’s work identified the direct role that infarct stiffness plays in controlling LV mechanics so as to inform the design of infarct restraint or stiffening therapies. Our current study developed a simple model to relate cardiac function to the features of the scar extracellular matrix rather than simply to stiffness through the use of a microstructure based constitutive model. The agreements that we see between our results and the results of the more complex finite element model presented by Fomovsky, in terms of increased longitudinal stiffness leading to increased SV give us confidence in our findings. A more recent study from the Holmes group has demonstrated that regional mechanics control collagen alignment [11]. In complement, we have demonstrated here that collagen alignment affects the scar mechanical properties and thus LV function. The model we have presented could be a simple, easy to use, analytical tool for studying how initial collagen alignment controls remodeling and infarct expansion through mechanical feedback.

While this model has offered much insight into the importance of collagen alignment in determining heart function, there are several limitations to the study. This model assumes a thin walled geometry which ignores the effects of transmural variation in stresses. While the choice of the thin-walled assumption may create additional error in the calculated stresses, it was deemed acceptable since the focus of the study was on identifying potential mechanisms rather than conducting detailed local stress analysis. Based on the fact that infarct scars are thinner than normal LV wall, we chose to model the infarct as a single layer with a disperse fiber alignment. In reality the infarct is composed of several layers of collagen fibers with a mean fiber orientation that varies through the wall thickness similar to the helical pattern of myocyte alignment in normal myocardium. This simplification in itself should have little effect on the results due to the thin wall assumption of the model. Further, the choice of a single fiber distribution simplified the fitting of the scar properties to experimental data. This model also neglects the effects of ventricular torsion, which plays an important role in systolic ejection [30].

In addition, the assumption that adjacent regions share deformations along the shared edges allowed for a simple method of enforcing material compatibility. On the downside, this means that the stress field is non-continuous. This assumption was made to capture the phenomenon of infarct coupling whereby increasing the stiffness of the infarct reduces the deformation of the neighboring regions. In reality this effect is much smaller in regions that are far from the infarct region than in the small border zones that surround the infarct with properties that transition from scar to normal, making it possible that the magnitude of the fiber orientation effects are slightly overstated by the model. Given that the properties in the normal regions should be independent of the infarct properties we believe that the mechanisms through which scar architecture influence SV identified by this model are correct. In whole we believe that the model is not oversimplified as it matches behavior reported both by Fomovsky, (improved SV with increased longitudinal stiffness) and by Bogen (reduced stiffness allowing for greater diastolic filling and contraction for small infarcts) [4,24].

The ground-up modeling approach we have chosen will allow us to refine the model in future work. While the collagen network is the primary determinant of infarct mechanics in the fibrotic scar, there are other structural proteins such as elastin and ECM modifications such as cross-linking that also play a role in LV mechanics [2]. Refinements of the constitutive model to include the effects of these ECM features would be an interesting direction for future work. In addition, the ischemic LV is highly inhomogeneous and further effort to model the border zones as well as the transmural variations in infarct properties would add to the physiological relevance of the model. Another future direction could examine the mechanics of the LV during the ischemic or inflammatory phases occurring immediately following MI. During these phases mechanical properties of the infarct are controlled less by collagen and more by the turnover of existing ECM and intracellular structural proteins such as titin as well as edema. The development of the collagen network and in turn the mechanical properties of the scar following this initial period are highly controlled by the expression of growth factors such as TGF-β and proteases such as MMPs [3,31]. Coupling the mechanical model to a dynamic model of protein regulation is one potential direction for future research. Another dynamic process that could be modeled in future work is the change in the orientation of the fibers that occur under load, which can dynamically change the mechanical properties of the tissue [32,33].

The post-MI LV is a complex and dynamic physiological environment and much work is ongoing to create highly accurate models. The goal of our paper was not to improve upon the accuracy of the existing numerical models, but to instead clarify the role that collagen plays in controlling infarct mechanics. Compared to some of the complex finite element models found in the literature, the simplicity of this model is actually an advantage as it focuses attention to the most important variables. This makes it a quick tool for identifying the key structural determinants of LV function post-MI. For example, the current simplicity of the model may make it a good choice for investigating many of the temporal changes that occur post-MI.

In summary, our model results showed that EF and SV are both optimized when fibers are aligned longitudinally, which further supports findings in the literature. The finding of an ideal fiber angle may aid in the development of diagnostic indices and treatment strategies based on determining fiber angle (perhaps through angle-sensitive MRI [34]) and directing it towards an optimal orientation through controlled remodeling. Since the model is based on the mechanical properties of actual infarct tissue it will also serve as an excellent starting point for creating multi-scale models that explore the interactions of genome, proteome, cell, tissue, and organ level changes in the post-MI remodeling process.

## Conclusions

A simple model of the post-MI LV was developed to demonstrate that a longitudinal alignment of collagen fibers in the scar region maximizes cardiac function. This improvement is the result of increased compliance in the circumferential direction which allows for greater diastolic filling and greater systolic contraction through the Frank-Starling mechanism.

## Appendix

### 1. Derivation of equilibrium equations

Many of the basic continuum mechanics equations used below can be found in Humphrey’s Cardiovascular Solid Mechanics text [25]. As described in the paper, there are six unknown deformation components. In order to solve for these six deformation components it is necessary to derive six independent equilibrium equations to relate stress to deformation. The internal forces in each region that balance the intraventricular pressure can be calculated from the Cauchy stress given in Eq. (9). If a matrix [***α***] is defined with rows $n_{i}^{T}$*a*_*i*_ and a matrix [***ξ***] is defined with rows *q*_*i*_ then Eq. (9) can be written as

(A1) $$\left[\xi \right]=\left[\alpha \right]\cdot \left[\sigma \right].$$

Similarly a matrix [***A***] can be defined with rows ***N***_***i***_*A*_*i*_ representing the areas in the undeformed configuration and a matrix [***Ξ***] with columns ***Q***_***i***_ representing the forces in the undeformed configuration. Both [***Ξ***] and [***A***] can be related to [***ξ***] and [***α***] respectively through the deformation gradient.

(A2) $$\left[\xi \right]=\left[\Xi \right]\cdot {\left[F\right]}^{T},$$

(A3) $$\left[\alpha \right]=\left[Α\right]\cdot {\left[F\right]}^{-1}.$$

In the undeformed configuration, it is assumed that the surface normals ***N***_***i***_ are aligned with the circumferential, longitudinal, and radial axes and that the internal forces in the undeformed configuration ***Q***_***i***_ are normal to the undeformed surface normals ***N***_***i***_ since pressure acts normal to surfaces. Thus,

(A4) $$\left[\Xi \right]=\left[\begin{array}{ccc} Q_{\theta \theta } & 0 & 0\\ 0 & Q_{\mathrm{zz}} & 0\\ 0 & 0 & 0 \end{array}\right],$$

and

(A5) $$\left[Α\right]=\left[\begin{array}{ccc} \mathrm{HT} & 0 & 0\\ 0 & \mathrm{LT} & 0\\ 0 & 0 & \mathrm{LH} \end{array}\right],$$

where, *H* is the undeformed, longitudinal height of the region, *L* is the undeformed, circumferential length, and *T* is the thickness of the region. Now solving Eq. (A2) in matrix form,

(A6) $$\left[\xi \right]=\left[\begin{array}{ccc} Q_{\theta \theta } & 0 & 0\\ 0 & Q_{\mathrm{zz}} & 0\\ 0 & 0 & 0 \end{array}\right]\left[\begin{array}{ccc} \lambda_{\theta } & 0 & 0\\ \kappa_{\mathrm{\theta z}} & \lambda_{z} & 0\\ 0 & 0 & \frac{1}{\lambda_{\theta }\lambda_{z}} \end{array}\right]=\left[\begin{array}{ccc} Q_{\theta \theta }\lambda_{\theta } & 0 & 0\\ Q_{\mathrm{zz}}\kappa_{\mathrm{\theta z}} & Q_{\mathrm{zz}}\lambda_{z} & 0\\ 0 & 0 & 0 \end{array}\right]=\left[\begin{array}{ccc} q_{\theta \theta } & 0 & 0\\ q_{\mathrm{z\theta}} & q_{\mathrm{zz}} & 0\\ 0 & 0 & 0 \end{array}\right],$$

it is seen that there are only 3 non-zero internal force components of [***ξ***]. Eq. (A3) can also be solved in matrix form for [*α*]

(A7) $$\left[\alpha \right]=\left[\begin{array}{ccc} \mathrm{HT} & 0 & 0\\ 0 & \mathrm{LT} & 0\\ 0 & 0 & \mathrm{LH} \end{array}\right]{\left[\begin{array}{ccc} \lambda_{\theta } & \kappa_{\mathrm{\theta z}} & 0\\ 0 & \lambda_{z} & 0\\ 0 & 0 & \frac{1}{\lambda_{\theta }\lambda_{z}} \end{array}\right]}^{-1}=\left[\begin{array}{ccc} \frac{\mathrm{HT}}{\lambda_{\theta }} & \frac{-\mathrm{HT}\kappa_{\mathrm{\theta z}}}{\lambda_{\theta }\lambda_{z}} & 0\\ 0 & \frac{\mathrm{LT}}{\lambda_{z}} & 0\\ 0 & 0 & \mathrm{LH}\lambda_{\theta }\lambda_{z} \end{array}\right].$$

Substituting Eq. (A7) into Eq. (A1) and assuming negligible radial stresses yields

(A8) $$\left[\xi \right]=\left[\begin{array}{ccc} \frac{\mathrm{HT}}{\lambda_{\theta }} & \frac{-\mathrm{HT}\kappa_{\mathrm{\theta z}}}{\lambda_{\theta }\lambda_{z}} & 0\\ 0 & \frac{\mathrm{LT}}{\lambda_{z}} & 0\\ 0 & 0 & \mathrm{LH}\lambda_{\theta }\lambda_{z} \end{array}\right]\left[\begin{array}{ccc} \sigma_{\theta \theta } & \sigma_{\mathrm{\theta z}} & 0\\ \sigma_{\mathrm{\theta z}} & \sigma_{\mathrm{zz}} & 0\\ 0 & 0 & 0 \end{array}\right]=\left[\begin{array}{ccc} \left(\sigma_{\theta \theta }-\sigma_{\mathrm{\theta z}}\frac{\kappa_{\mathrm{\theta z}}}{\lambda_{z}}\right)\frac{\mathrm{HT}}{\lambda_{\theta }} & \left(\sigma_{\mathrm{\theta z}}-\sigma_{\mathrm{zz}}\frac{\kappa_{\mathrm{\theta z}}}{\lambda_{z}}\right)\frac{\mathrm{HT}}{\lambda_{\theta }} & 0\\ \sigma_{\mathrm{\theta z}}\frac{\mathrm{LT}}{\lambda_{z}} & \sigma_{\mathrm{zz}}\frac{\mathrm{LT}}{\lambda_{z}} & 0\\ 0 & 0 & 0 \end{array}\right].$$

Relating the result of Eq. (A8) with the components of [***ξ***] as given by Eq. (A6) provides a system of 4 equations

(A9.a) $$q_{\theta \theta }=\left(\sigma_{\theta \theta }-\sigma_{\mathrm{z\theta}}\frac{\kappa_{\mathrm{\theta z}}}{\lambda_{z}}\right)\frac{\mathrm{HT}}{\lambda_{\theta }},$$

(A9.b) $$0=\left(\sigma_{\mathrm{\theta z}}-\sigma_{\mathrm{zz}}\frac{\kappa_{\mathrm{\theta z}}}{\lambda_{z}}\right)\frac{\mathrm{HT}}{\lambda_{\theta }},$$

(A9.c) $$q_{\mathrm{z\theta}}=\sigma_{\mathrm{\theta z}}\frac{\mathrm{LT}}{\lambda_{z}},$$

(A9.d) $$q_{\mathrm{zz}}=\sigma_{\mathrm{zz}}\frac{\mathrm{LT}}{\lambda_{z}}.$$

From Eq. (A9.b), it is seen that

(A10) $$\sigma_{\mathrm{\theta z}}=\sigma_{\mathrm{zz}}\frac{\kappa_{\mathrm{\theta z}}}{\lambda_{z}}.$$

Combining this fact with Eq. (A9.c) and Eq. (A9.d) yields the following relationship between force and deformation

(A11) $$\frac{q_{\mathrm{z\theta}}}{q_{\mathrm{zz}}}=\frac{\kappa_{\mathrm{\theta z}}}{\lambda_{z}}.$$

Now that relationships between force, stress, and deformation have been established for individual regions it is necessary to examine the four region model with forces and deformations being shared as described in Figure 3. In order to determine the magnitudes of the internal force vectors described in Eq. (11) it is necessary to examine the components of these vectors again.

From Eq. (A6) it is seen that

(A12.a) $$q_{\theta }=q_{\theta \theta }i_{\theta },$$

(A12.b) $$q_{z}=q_{\mathrm{z\theta}}i_{\theta }+q_{\mathrm{zz}}i_{z},$$

where ***i***_***θ***_ and ***i***_***z***_ are unit vectors. From Eq. (A12.a) it is seen that

(A13) $$||q_{\theta }||=q_{\theta \theta },$$

which allows for Eq. (11.a) to be rewritten as

(A14) $$q_{\theta \theta }={q_{\theta \theta }}^{I}+{q_{\theta \theta }}^{\mathrm{III}}={q_{\theta \theta }}^{\mathrm{II}}+{q_{\theta \theta }}^{S}=\mathrm{Prh}.$$

Substituting Eq. (A9.a) for each region into Eq. (A14) yields 2 equilibrium equations

(A15.a) $$\left({\sigma_{\theta \theta }}^{I}-{\sigma_{\mathrm{z\theta}}}^{I}\frac{{\kappa_{\mathrm{\theta z}}}^{I}}{{\lambda_{z}}^{I}}\right)\frac{H^{I}T^{I}}{{\lambda_{\theta }}^{I}}+\left({\sigma_{\theta \theta }}^{\mathrm{III}}-{\sigma_{\mathrm{z\theta}}}^{\mathrm{III}}\frac{{\kappa_{\mathrm{\theta z}}}^{\mathrm{III}}}{{\lambda_{z}}^{\mathrm{III}}}\right)\frac{H^{\mathrm{III}}T^{\mathrm{III}}}{{\lambda_{\theta }}^{\mathrm{III}}}=\mathrm{Prh},$$

(A15.b) $$\left({\sigma_{\theta \theta }}^{\mathrm{II}}-{\sigma_{\mathrm{z\theta}}}^{\mathrm{II}}\frac{{\kappa_{\mathrm{\theta z}}}^{\mathrm{II}}}{{\lambda_{z}}^{\mathrm{II}}}\right)\frac{H^{\mathrm{II}}T^{\mathrm{II}}}{{\lambda_{\theta }}^{\mathrm{II}}}+\left({\sigma_{\theta \theta }}^{S}-{\sigma_{\mathrm{z\theta}}}^{S}\frac{{\kappa_{\mathrm{\theta z}}}^{S}}{{\lambda_{z}}^{S}}\right)\frac{H^{S}T^{S}}{{\lambda_{\theta }}^{S}}=\mathrm{Prh}.$$

The vector ***q***_***z***_ can be written in polar notation as

(A16) $$q_{z}=||q_{z}||\sin\left(\varphi \right)i_{\theta }+||q_{z}||\cos\left(\varphi \right)i_{z}.$$

where

(A17) $$\varphi =\mathrm{atan}\left(\frac{q_{\mathrm{z\theta}}}{q_{\mathrm{zz}}}\right).$$

Substituting Eq. (A11) into Eq. (A17) yields

(A18) $$\varphi =\mathrm{atan}\left(\frac{\kappa_{\mathrm{\theta z}}}{\lambda_{z}}\right).$$

Equating Eq. (A16) with Eq. (A12.b) gives

(A19.a) $$q_{\mathrm{z\theta}}=||q_{z}||\sin\left(\varphi \right),$$

(A19.b) $$q_{\mathrm{zz}}=||q_{z}||\cos\left(\varphi \right),$$

Solving Eq. (11.b) for the magnitude and equating with Eq. (A19) yields

(A20.a) $$||q_{z}||=\frac{q_{\mathrm{z\theta}}}{\sin\left(\varphi \right)}=\frac{{q_{\mathrm{z\theta}}}^{I}}{\sin\left(\varphi^{I}\right)}+\frac{{q_{\mathrm{z\theta}}}^{\mathrm{II}}}{\sin\left(\varphi^{\mathrm{II}}\right)}=\frac{{q_{\mathrm{z\theta}}}^{\mathrm{III}}}{\sin\left(\varphi^{\mathrm{III}}\right)}+\frac{{q_{\mathrm{z\theta}}}^{S}}{\sin\left(\varphi^{\mathrm{IV}}\right)}=\mathrm{\pi P}r^{2},$$

(A20.b) $$||q_{z}||=\frac{q_{\mathrm{zz}}}{\cos\left(\varphi \right)}=\frac{{q_{\mathrm{zz}}}^{I}}{\cos\left(\varphi^{I}\right)}+\frac{{q_{\mathrm{zz}}}^{\mathrm{II}}}{\cos\left(\varphi^{\mathrm{II}}\right)}=\frac{{q_{\mathrm{zz}}}^{\mathrm{III}}}{\cos\left(\varphi^{\mathrm{III}}\right)}+\frac{{q_{z}}^{S}}{\cos\left(\varphi^{\mathrm{IV}}\right)}=\mathrm{\pi P}r^{2}.$$

Substitution of Eq. (A9.c) for each region into Eq. (A20.a) gives 2 more equilibrium equations

(A21.a) $${\sigma_{\mathrm{\theta z}}}^{I}\frac{L^{I}T^{I}}{{\lambda_{z}}^{I}\sin\left(\varphi^{I}\right)}+{\sigma_{\mathrm{\theta z}}}^{\mathrm{II}}\frac{L^{\mathrm{II}}T^{\mathrm{II}}}{{\lambda_{z}}^{\mathrm{II}}\sin\left(\varphi^{\mathrm{II}}\right)}=\mathrm{\pi P}r^{2},$$

(A21.b) $${\sigma_{\mathrm{\theta z}}}^{\mathrm{III}}\frac{L^{\mathrm{III}}T^{\mathrm{III}}}{{\lambda_{z}}^{\mathrm{III}}\sin\left(\varphi^{\mathrm{III}}\right)}+{\sigma_{\mathrm{\theta z}}}^{S}\frac{L^{S}T^{S}}{{\lambda_{z}}^{S}\sin\left(\varphi^{S}\right)}=\mathrm{\pi P}r^{2}.$$

Substitution of Eq. (A9.d) for each region into Eq. (A20.b) gives the last 2 equilibrium equations

(A22.a) $${\sigma_{\mathrm{zz}}}^{I}\frac{L^{I}T^{I}}{{\lambda_{z}}^{I}\cos\left(\varphi^{I}\right)}+{\sigma_{\mathrm{zz}}}^{\mathrm{II}}\frac{L^{\mathrm{II}}T^{\mathrm{II}}}{{\lambda_{z}}^{\mathrm{II}}\cos\left(\varphi^{\mathrm{II}}\right)}=\mathrm{\pi P}r^{2},$$

(A22.b) $${\sigma_{\mathrm{zz}}}^{\mathrm{III}}\frac{L^{\mathrm{III}}T^{\mathrm{III}}}{{\lambda_{z}}^{\mathrm{III}}\cos\left(\varphi^{\mathrm{III}}\right)}+{\sigma_{\mathrm{zz}}}^{S}\frac{L^{S}T^{S}}{{\lambda_{z}}^{S}\cos\left(\varphi^{S}\right)}=\mathrm{\pi P}r^{2}.$$

Collecting Eq. (A15), Eq. (A21), and Eq. (A22) and noting the shared lengths, heights, and deformations as shown in Figure 1 produces the final set of equilibrium equations.

(A23.a) $$\left({\sigma_{\theta \theta }}^{I}-{\sigma_{\mathrm{z\theta}}}^{I}\frac{{\kappa_{\mathrm{\theta z}}}^{R}}{{\lambda_{z}}^{R}}\right)\frac{H^{R}T^{I}}{{\lambda_{\theta }}^{R}}+\left({\sigma_{\theta \theta }}^{\mathrm{III}}-{\sigma_{\mathrm{z\theta}}}^{\mathrm{III}}\frac{{\kappa_{\mathrm{\theta z}}}^{S}}{{\lambda_{z}}^{S}}\right)\frac{H^{S}T^{\mathrm{III}}}{{\lambda_{\theta }}^{R}}=\mathrm{Prh},$$

(A23.b) $$\left({\sigma_{\theta \theta }}^{\mathrm{II}}-{\sigma_{\mathrm{z\theta}}}^{\mathrm{II}}\frac{{\kappa_{\mathrm{\theta z}}}^{R}}{{\lambda_{z}}^{R}}\right)\frac{H^{R}T^{\mathrm{II}}}{{\lambda_{\theta }}^{S}}+\left({\sigma_{\theta \theta }}^{S}-{\sigma_{\mathrm{z\theta}}}^{S}\frac{{\kappa_{\mathrm{\theta z}}}^{S}}{{\lambda_{z}}^{S}}\right)\frac{H^{S}T^{S}}{{\lambda_{\theta }}^{S}}=\mathrm{Prh},$$

(A23.c) $${\sigma_{\mathrm{\theta z}}}^{I}\frac{L^{R}T^{I}}{{\lambda_{z}}^{R}}+{\sigma_{\mathrm{\theta z}}}^{\mathrm{II}}\frac{L^{S}T^{\mathrm{II}}}{{\lambda_{z}}^{R}}=\mathrm{\pi P}r^{2}\sin\left(\varphi^{R}\right),$$

(A23.d) $${\sigma_{\mathrm{\theta z}}}^{\mathrm{III}}\frac{L^{R}T^{\mathrm{III}}}{{\lambda_{z}}^{S}}+{\sigma_{\mathrm{\theta z}}}^{\mathrm{IV}}\frac{L^{S}T^{S}}{{\lambda_{z}}^{S}}=\mathrm{\pi P}r^{2}\sin\left(\varphi^{S}\right).$$

(A23.e) $${\sigma_{\mathrm{zz}}}^{I}\frac{L^{R}T^{I}}{{\lambda_{z}}^{R}}+{\sigma_{\mathrm{zz}}}^{\mathrm{II}}\frac{L^{S}T^{\mathrm{II}}}{{\lambda_{z}}^{R}}=\mathrm{\pi P}r^{2}\cos\left(\varphi^{R}\right),$$

(A23.f) $${\sigma_{\mathrm{zz}}}^{\mathrm{III}}\frac{L^{R}T^{\mathrm{III}}}{{\lambda_{z}}^{S}}+{\sigma_{\mathrm{zz}}}^{S}\frac{L^{S}T^{S}}{{\lambda_{z}}^{S}}=\mathrm{\pi P}r^{2}\cos\left(\varphi^{S}\right),$$

where

(A24.a) $$\varphi^{R}=\mathrm{atan}\left(\frac{{\kappa_{\mathrm{\theta z}}}^{R}}{{\lambda_{z}}^{R}}\right),$$

(A24.b) $$\varphi^{S}=\mathrm{atan}\left(\frac{{\kappa_{\mathrm{\theta z}}}^{S}}{{\lambda_{z}}^{S}}\right).$$

This set of 6 independent equilibrium equations can be used in conjunction with the constitutive equations to solve for the 6 independent deformation components, $\lambda_{\theta }^{R},\lambda_{\theta }^{S},\lambda_{z}^{R},\lambda_{z}^{S},\kappa_{\mathrm{\theta z}}^{R}\;\mathrm{and}\;\kappa_{\mathrm{\theta z}}^{S}$.

## Competing interests

The authors declare that they have no competing interests.

## Authors’ contributions

AV and HCH designed the study and developed the model. AV implemented the numerical solution techniques and analyzed the data. Both authors participated in the preparation of the manuscript and acknowledge that they have read and approved the final version of the manuscript.
